# Prognostic Value of Gamma-Glutamyltransferase in Male Patients With Idiopathic Pulmonary Arterial Hypertension

**DOI:** 10.3389/fcvm.2020.580908

**Published:** 2020-10-23

**Authors:** Gang-Hua Lu, Su-Gang Gong, Chao Li, Qin-Hua Zhao, Rong Jiang, Ci-Jun Luo, Lan Wang, Rui Zhang

**Affiliations:** ^1^Tongji University School of Medicine, Shanghai, China; ^2^Department of Cardio-Pulmonary Circulation, Shanghai Pulmonary Hospital, Tongji University School of Medicine, Shanghai, China

**Keywords:** biomarkers, gamma-glutamyltransferase, gender difference, idiopathic pulmonary arterial hypertension, prognosis

## Abstract

**Background:** The elevated gamma-glutamyltransferase (GGT) activity is regarded as an indicator of cardiovascular disease, with males having higher values than females. The greater incidence of idiopathic pulmonary arterial hypertension (IPAH) is observed in women, whereas prognosis is poor in men. The present study aims to investigate the potential association of GGT on male patients.

**Methods:** Serum GGT levels were measured in 338 consecutive adult IPAH patients, who underwent bone morphogenetic protein receptor type 2 (*BMPR2*) genetic counseling, and matched with healthy subjects by sex and age. The followed interval was 48 ± 34 months.

**Results:** Increased serum GGT levels were more common in patients with IPAH than controls (*p* < 0.001). GGT values were significantly higher in male patients than those of females (*p* < 0.001). Compared with female patients with *BMPR2* mutation, GGT level in male patients with *BMPR2* mutation was further increased (*p* = 0.002). Higher GGT levels were associated with worse hemodynamics and Nterminal pro B-type natriuretic peptide in all patients. However, males with a GGT concentration ≥ 53 U/L had a worse survival than those of females. Contrarily, if GGT concentration <53 U/L, there was no survival difference between male and female patients. After adjustment for relevant variables of clinical features and hemodynamics, baseline higher GGT levels remained increased risks of all-cause mortality in males rather than females. During rehospitalization follow-up, male patients still had significantly higher values of GGT than females.

**Conclusions:** Increased GGT levels were correlated with *BMPR2* mutation, hemodynamic dysfunction, and poor outcomes in male patients with IPAH. Further studies are needed to explain the origin of abnormal GGT and its potential pathogenesis in men.

## Introduction

Patients with pulmonary arterial hypertension (PAH) have a poor prognosis due to progressive pulmonary vascular remodeling, which leads to right ventricular (RV) overload and right heart failure without effective treatment (1). There is a famous sex paradox in PAH: females have more predominance in developing PAH than males, yet have better survival than males (2). Complex sex hormone signaling or processing pathways in the pathology of idiopathic PAH (IPAH) have been observed, such as impacts of different sex hormones in the RV function, bone morphogenetic protein receptor type 2 (*BMPR2*) mutation, oxidative stress and autoimmunity (3–6). However, the reason for the inconsistency remains unclear.

Gamma-glutamyltransferase (GGT) is a liver enzyme located on most cell membranes and organ tissues, especially hepatocytes (7). Although GGT activity is determined by genes and age, there is still a remarkable sex difference that females have lower value than males in normal physiology (7, 8). Young and pregnant females have decreased GGT activity, while GGT activity in postmenopausal females and those of taking oral contraceptives is significantly increased, closed to the levels of males (8, 9). There are different normal reference value ranges in some countries, for example, men ≤ 50 U/L and women ≤ 40 U/L in Canada (10) and Finland (11); men ≤ 28 U/L and women ≤ 18 U/L in Australia (12); men and women ≤ 50 U/L in Korea (13); men and women 12–58 U/L in Japan (14). However, in clinical practice in China, different sex had the same reference value range of GGT (normal range ≤ 38 U/L). And so far, it was unclear whether GGT involved in the pathology of PAH. As a transaminase, GGT generally plays a role in the catabolism of extracellular glutathione, a major antioxidant protecting cells against oxidants (8). Serum GGT has been regarded as a biomarker in cardiovascular disease (CVD) with great sex difference (15–17). For example, Wannamethee et al. have reported that elevated serum GGT activity was a predictor of increased CVD mortality in British men, particularly in those with age <55 years (15). In a German study, increased GGT level was associated with risk of cardiometabolic mortality in men, but not in women (16). However, a Japanese study showed that the relationship between levels of GGT and CVD mortality was not evident in male patients (17). Taken together, the above information implicated that serum GGT was an important indicator, likely to provide the basis for risk assessments or individual treatments.

Although there are some biomarkers used for diagnosis and prediction of IPAH, few of them are about sex differences (18). Until now, it is limited information on serum GGT alteration in patients with IPAH, especially in male patients. Therefore, the objectives of this study were to determine whether (a) GGT levels were further increased in male patients; (b) abnormalities of GGT activities were related to hemodynamic dysfunction and clinical characters; (c) male patients with *BMPR2* mutation had higher GGT levels than those of females; (d) serum GGT could be a predictor for prognosis and clinical outcomes in male patients with IPAH.

## Materials and Methods

### Study Subjects and Design

A total of 338 consecutive patients with IPAH (age ≥ 18 years at diagnosis), who underwent *BMPR2* gene detection at their first right heart catheterization (RHC) were enrolled in this study between July 2010 and December 2018. Control subjects of the same number were chosen from a group of healthy volunteers. The median age of the control participates was 41 years (range: 36–47), and the ratio of females to males was 2.2:1. The diagnosis of IPAH was based on a standard criteria: a mean pulmonary artery pressure (mPAP) ≥ 25 mmHg, pulmonary vascular resistance (PVR) > 3 Woods units, and pulmonary artery wedge pressure (PAWP) ≤ 15 mmHg at rest (19, 20). Patients were excluded if they had a definite disease associated with connective-tissue disease and congenital heart disease, and portopulmonary hypertension, and chronic pulmonary thromboembolism and pulmonary hypertension caused by left heart disease, and lung diseases and/or hypoxaemia (19, 20). Patients were also excluded with smoking, alcohol abuse, hypercholesterolaemia, non-alcoholic fatty liver disease, hypertension, atherosclerosis, stroke or type 2 diabetes mellitus. We also did not consider the participants who had taken cyclooxygenase (COX) inhibitors, such as non-steroidal anti-inflammatory medications, aspirin or COX-2 inhibitors within 14 days of blood samples collection (20).

Cardiopulmonary exercise testing was performed on an electromagnetically braked cycle ergometer (Master Screen CPX, Jaeger crop, Hoechberg, Germany) according to the American Thoracic Society and American College of Chest Physicians statement (21). Patients underwent a standardized transthoracic echocardiographic examination by GE Vivid 7 Ultrasound (GE Vingmed Ultrasound, Horten, Norway). All echocardiographic parameters were performed under guidelines of American Society of Echocardiography recommendations within 2 days of RHC measurement (22).

We followed these patients for a mean 48 ± 34 months after enrolled in this study. The major endpoint of the follow-up was regarded as the all-cause mortality. During the follow-up interval, 239 patients were hospitalized again, who had at least one lab examination including GGT and N-terminal pro B-type natriuretic peptide (NT-proBNP), et al. The average duration of time was 20 ± 22 months in males and 22 ± 20 months in females, respectively, between the first time in hospital and rehospitalization. The study was conducted according to the principles of the Declaration of Helsinki and was approved by the Shanghai Pulmonary Hospital Ethics Committee (Number: K18-025). Written informed consent was obtained from all participants.

### Blood Sampling and Serum GGT Measurement

After overnight fasting, blood samples were drawn from peripheral venous blood, collected into serum separating tubes and randomly ordered for testing at 37°C. All analyses were performed at the biochemistry laboratory of Shanghai Pulmonary Hospital by ADVIA 2400 Clinical Chemistry System (Siemens Healthcare) using Siemens reagent 02011954. The method for measurement of GGT enzyme activity was standardized according to the International Federation of Clinical Chemistry and Laboratory Medicine (IFCC) reference measurement procedures (23). The intra-assay coefficient of variation was estimated at 1.9%. The laboratory technicians were blinded to do and the normal reference values were 0–38 U/L for men and women. Blood samples were obtained within 2 days prior to RHC.

### Statistical Analysis

Data were expressed as numbers, percentages, medians with corresponding 25 and 75th percentiles [interquartile range (IQR)] or means with corresponding standard deviations. Comparisons of parameters between 2 groups were taken by Mann-Whitney *U*-test or unpaired Student *t*-test for continuous data. The proportions for categorical data were compared with the Pearson Chi-square test or Fisher's exact test as appropriate. A frequency division histogram was shown in *BMPR2* non-mutation and mutation groups, comparisons of GGT activity between male and female patients were taken by unpaired Student *t*-test. The linearity of relationships between the GGT and hemodynamic variables, NTproBNP as well as right atrial area (RA area) was investigated by Spearman's test.

To compare the prognostic values of GGT, NT-ProBNP, 6-minute walking distance (6MWD) and selected hemodynamic parameters, receiver operating characteristic (ROC) curves were generated, and the areas under the curves (AUCs) were calculated. Survival analyses were performed using the KaplanMeier method and were compared using the log-rank test, adjusted by age. The prognostic values of the parameters were tested in univariate Cox proportionalhazards regression analysis, such as demographic, clinical data and hemodynamic variables. Continuous variables were transferred into categorical variables by their cut-off values. Variables were all incorporated into a forward stepwise multivariable Cox proportional hazards model, if considered to be confounders in the univariate analyses or have clinical importance. A value of *p* < 0.05 was considered statistically significant. Statistical analyses were performed with SPSS 14.0 statistical software package (Statistical Package for Social Science, Chicago, IL, USA).

## Results

### GGT Activity and Clinical Characteristics

**Figure 1:**
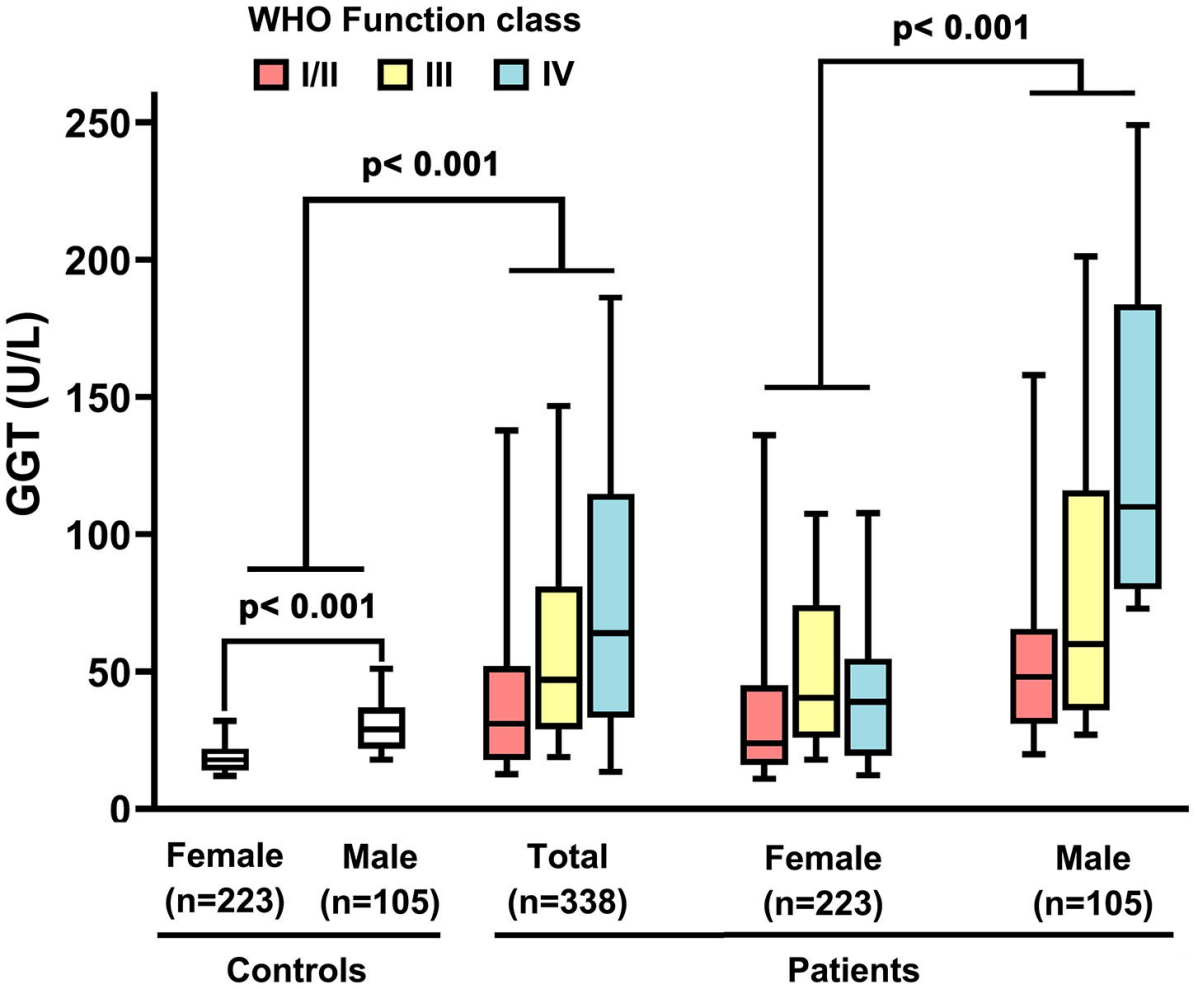
Serum gamma-glutamyltranspeptidase (GGT) concentrations in idiopathic pulmonary arterial hypertension patients, controls and different sex, associated with World Health Organization (WHO) functional class. The line through the center of the box represents the mean.

**Figure 2:**
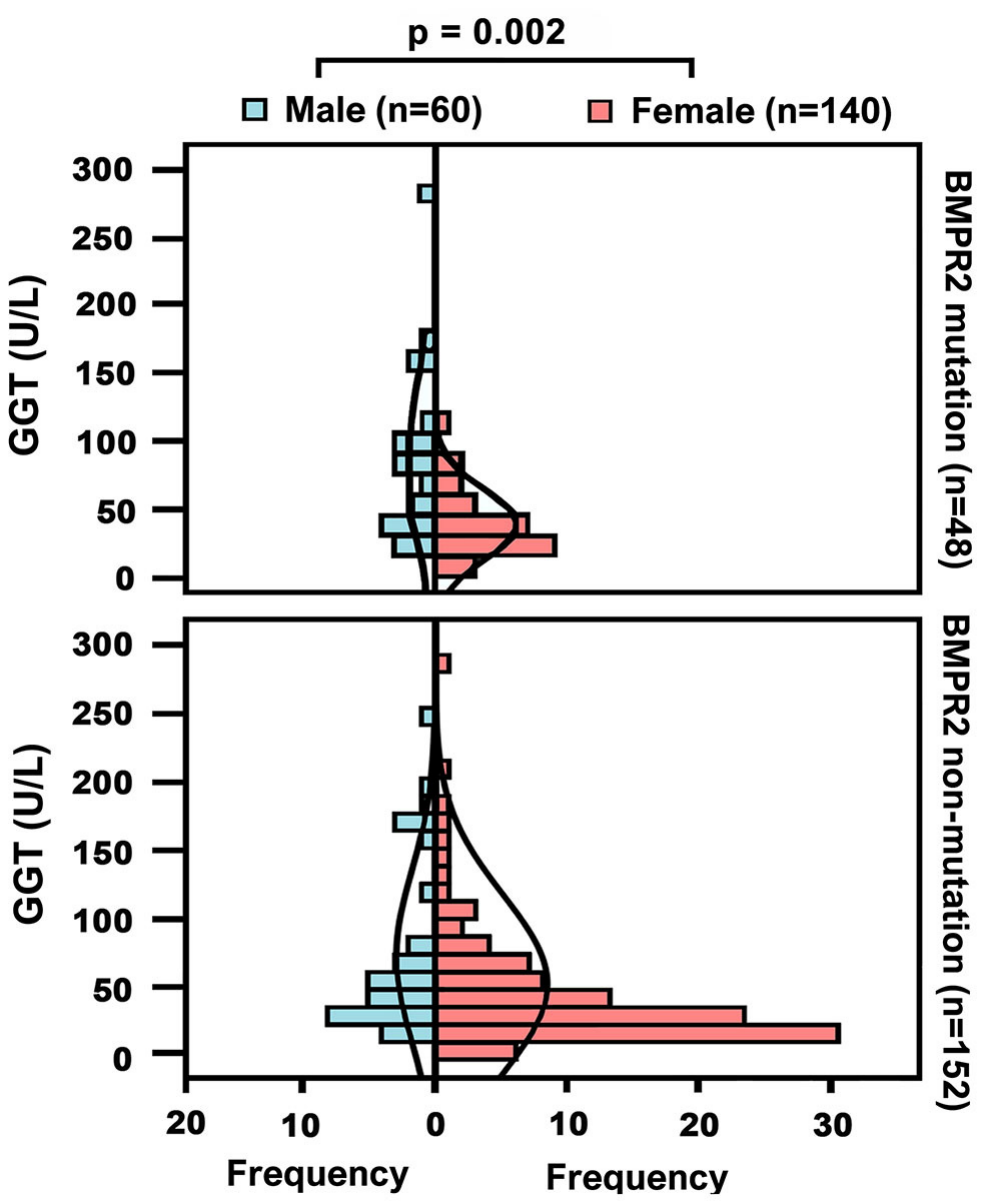
Baseline gamma-glutamyltranspeptidase (GGT) concentrations in different sex groups or patients with bone morphogenetic protein receptor type2 (BMPR2) mutation and non-mutation.

Demographic and biochemical characteristics of IPAH patients are summarized in Table 1. Among 338 IPAH patients, 105 (31%) patients were males, and the male/female ratio was 1: 2.2. The male patients had an older mean age (44 ± 19 years) than female patients (39 ± 14 years, *p* = 0.003, Table 1). In healthy controls, the activity of GGT in men was higher than that in women (31.8 ± 14.3 vs. 20.2 ±11 U/L, *p* < 0.001, Figure 1). Accordingly, the serum GGT activity was greatly increased in total IPAH patients compared with all control subjects (64.2 ± 67.1 vs. 23.8 ±13.3 U/L, *p* < 0.001, Figure 1). Also, serum GGT activity was significantly higher in male patients than that in females (89.3 ± 82.2 vs. 52.6 ± 55.4 U/L, *p* < 0.001). The GGT concentration in men was markedly reduced in patients with World Health Organization Function Class (WHO FC) I/II (50.1 ± 39.3 U/L), compared with those with WHO FC III (63.2 ± 56.2 U/L, *p* = 0.04) or IV (99.9 ± 88.8 U/L, *p* = 0.01), respectively. However, there was no significant difference of GGT in regard to WHO FC severity in female patients. Among 48 patients with *BMPR2* mutation, the mutation proportion of male patients (35%) was significantly higher than that of female patients (19%, *p* = 0.017, Table 1). As shown in Figure 2, in patients with *BMPR2* mutation, GGT levels were greatly increased in male patients than female patients (84.0 ± 70.2 vs. 35.5 ± 21.2 U/L, *p* = 0.002). However, GGT levels in patients with non-*BMPR2* mutation were not any different between males and females.

**Table 1:** Baseline characteristics of patients with IPAH.

**Variables**	**Control (*n* = 338)**	**Total (*n* = 338)**	**Male (*n* = 105)**	**Female (*n* = 233)**	*P-*value^*^
Age, years	41 ± 9	40 ± 16	44 ± 19	39 ± 14	0.003
BMI, kg/m^2^	22 (20–24)	22 (20–24)	22 (20–24)	22 (20–24)	0.834
6MWD, meters^†^	-	380 (305–445)	398 (308–471)	374 (305–440)	0.112
WHO FC, *n* (%)					0.586
Class I/II	-	106 (31)	32 (30)	74 (32)	
Class III	-	210 (62)	64 (61)	146 (62)	
Class IV	-	22 (7)	9 (9)	13 (6)	
*BMPR2* mutation, *n* (%)^‡^	*-*	48 (24)	21 (35)	27 (19)	0.017
NT-proBNP, ng/L	-	1157 ± 1212	1339 ± 1532	1075 ± 1031	0.113
**Liver function**					
GGT, U/L	23.8 ± 13.3^#^	64.2 ± 67.1	89.3 ± 82.2	52.6 ± 55.4	<0.001
ALT, U/L	22.7 ± 16.2	29.4 ± 22.8	31.3 ± 19.9	28.6 ± 24.0	0.286
AST, U/L	20.0 ± 5.5	28.8 ± 20.0	28.8 ± 16.9	28.8 ± 21.3	1.000
**Renal function**					
Creatinine, μmol/L	61.2 ± 12.4	68.7 ± 24.0	74.5 ± 33.3	61.9 ± 14.1	<0.001
BUN, mmol/L	4.7 ± 1.2	5.5 ± 2.1	6.5 ± 2.6	5.1 ± 1.7	<0.001
UA, μmol/L	285.0 ± 74.2^#^	409.4 ± 125.7	446.6 ± 131.3	393.4 ± 120.0	0.001
**Hemodynamic variables**					
mRAP, mmHg	-	7.0 ± 5.2	7.4 ± 5.7	6.8 ± 5.0	0.398
mPAP, mmHg	-	58.2 ± 14.7	60.5 ± 16.6	57.1 ± 13.6	0.044
PAWP, mmHg	-	7.6 ± 3.3	8.0 ± 3.1	7.4 ± 3.3	0.031
CI, L/min/m^2^	-	2.6 ± 0.8	2.5 ± 0.8	2.6 ± 0.8	0.486
PVR, Wood units	-	13.8 ± 6.5	15.3 ± 6.5	12.6 ± 6.6	0.034
S_V_O_2_, %		63 (57–69)	62 (56–70)	64 (57–69)	0.278
**Cardiopulmonary exercise testing^§^**					
Peak VO_2_, mL/min/kg	-	13 (10–16)	13 (10–17)	13 (11–15)	0.784
VE/VCO_2_ slope	-	56 ± 33	60 ± 29	54 ± 36	0.336
**Echocardiography**					
RA area, cm^2^^∥^	-	25 ± 13	30 ± 15	23 ± 11	0.001
**PAH-Specific therapies**, ***n*** **(%)**					0.394
ERA	-	39 (13)	16 (17)	23 (11)	
PDE5-i	-	141 (46)	46 (47)	95 (45)	
Prostanoids	-	17 (5)	4 (4)	13 (6)	
Combination therapy	-	112 (36)	31 (32)	81 (38)	

*Data were presented as numbers of patients (n, %), mean ± standard deviations or median (interquartile range)*.

* *Comparison between male and female*.

# *Comparison between controls and total patients, # represented p < 0.05*.

† *Data on 6MWD was available from 275 individuals*.

‡ *Data on BMPR2 mutation measurement was available from 200 individuals*.

§ *Data on cardiopulmonary exercise testing was available from 100 individuals*.

∥ *RA area was measured in 222 individuals*.

*ALT, glutamate pyruvate transaminase; AST, glutamic oxalacetic transaminase; BMI, body mass index; BMPR2, bone morphogenetic protein receptor type 2; BUN, blood urea nitrogen; CI, cardiac index; ERA, endothelin receptor antagonist; GGT, gamma-glutamyltranspeptidase; IPAH, idiopathic pulmonary arterial hypertension; mPAP, mean pulmonary arterial pressure; PAWP, mean pulmonary artery wedge pressure; mRAP, mean right atrial pressure; 6MWD, 6minute walking distance; NT-proBNP, N-terminal pro B-type natriuretic peptide; PDE5-i, phosphodiesterase type 5 inhibitors; Peak VO_2_, peak oxygen uptake; PVR, pulmonary vascular resistance; RA area, right atrial area; S_v_O_2_, mixed venous oxygen saturation; UA, uric acid; VE/VCO_2_ slope, ventilatory equivalent for carbon dioxide slope; WHO FC, World Health Organization functional class*.

### Correlation of GGT Activity With Hemodynamic Variables in Males and Females

**Figure 3:**
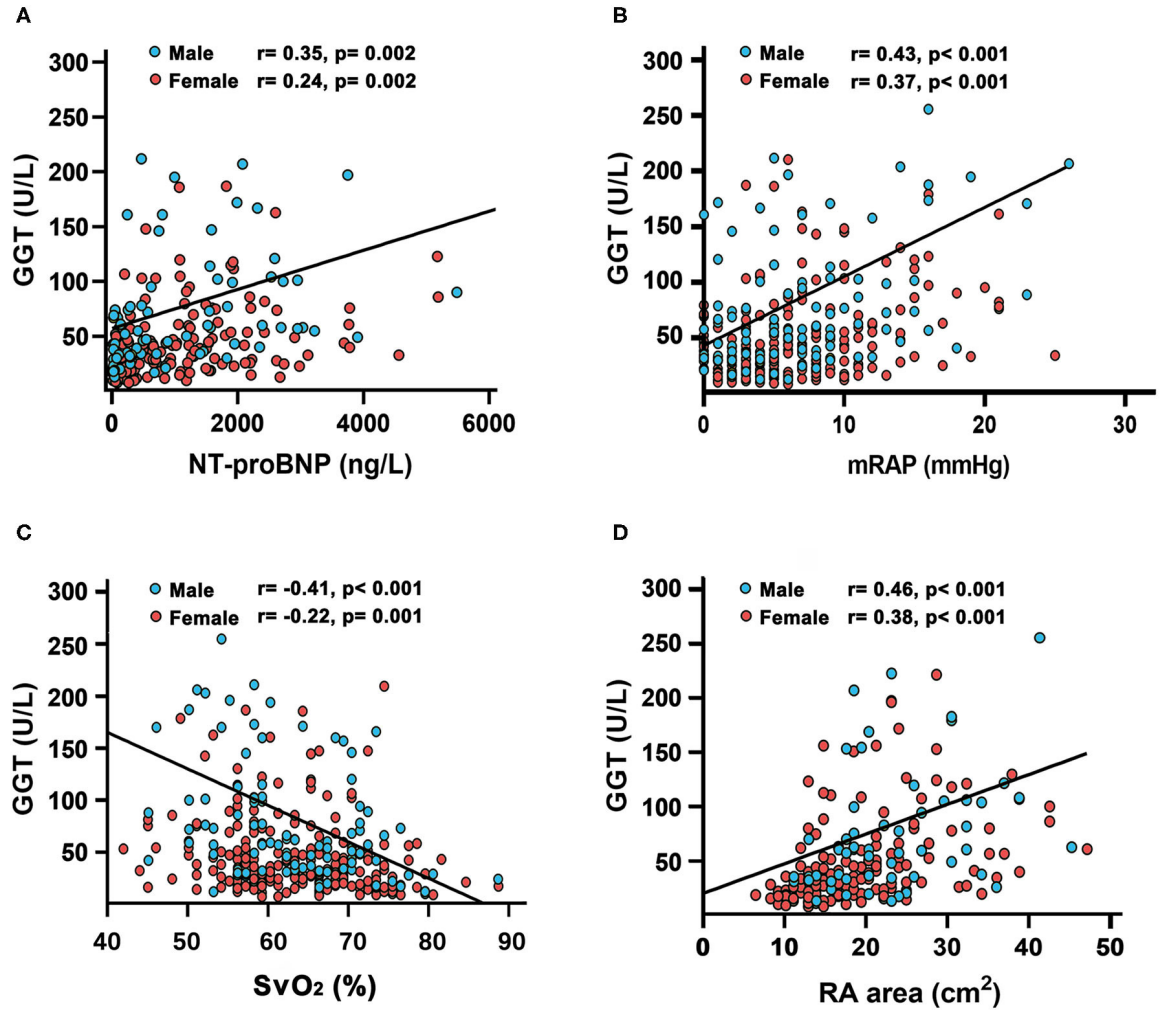
Relationship between gamma-glutamyltranspeptidase (GGT) concentrations with **(A)** N-terminal pro B-type natriuretic peptide (NT-proBNP); **(B)** mean right atrial pressure(mRAP); **(C)** mixed venous oxygen saturation (S_v_O_2_) and **(D)** right atrial (RA) area in patients with idiopathic pulmonary arterial hypertension.

Compared with female patients, male patients had worse hemodynamic status, such as higher mPAP, PVR, and RA area (Table 1). Baseline serum GGT were positively associated with NT-proBNP (*r* = 0.35, *p* = 0.002 in male; *r* = 0.24, *p* = 0.002 in female), mean right atrial pressure (mRAP) (*r* = 0.43, *p* < 0.001 in male; *r* = 0.37, *p* < 0.001 in female), and RA area (*r* = 0.46, *p* < 0.001 in male; *r* = 0.38, *p* < 0.001 in female, Figures 3A,B,D), and negatively associated with mixed venous oxygen saturation (S_V_O_2_) (*r* = −0.41, *p* < 0.001 in male; *r* = −0.22, *p* = 0.001 in female, Figure 3C). The correlations between GGT and other hemodynamic variables, 6MWD, creatinine, blood urea nitrogen (BUN), Uric acid (UA), age and body mass index (BMI) are shown in Supplemental Figures 1–9.

### Survival Analysis

**Figure 4:**
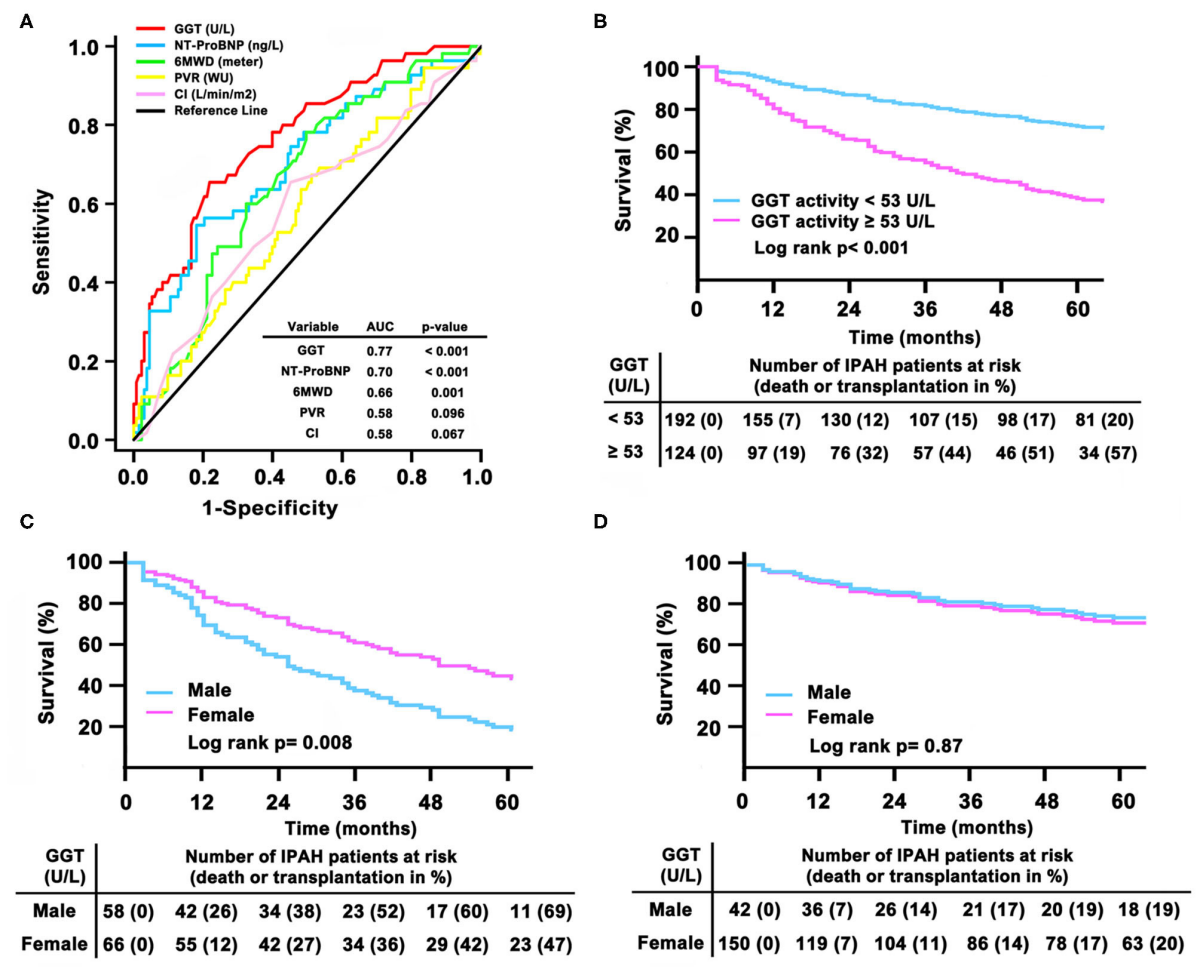
Gamma-glutamyltranspeptidase (GGT) in relation to other markers of adverse prognosis by **(A)** receiver operation characteristic analysis; **(B)** Kaplan– Meier survival curves according to the baseline cut-off serum GGT activity in total patients; **(C)** survival in different sex in GGT activity ≥ 53 U/L group and **(D)** in GGT activity <53 U/L group. AUC, area under the curves; CI, cardiac index; IPAH, idiopathic pulmonary arterial hypertension; 6MWD, 6-minute walking distance; NT-proBNP, N-terminal pro B-type natriuretic peptide; PVR, pulmonary vascular resistance.

During the follow-up period, no patients received lung or heart-lung transplant. In this study, we found male patients had lower survival rate than females (logrank test, *p* = 0.001, Supplemental Figure 10). ROC curve demonstrated that GGT [AUC 0.77; 95% confidence interval (CI) 0.7–0.84] was numerically superior to NT-proBNP (AUC 0.7; 95% CI 0.62–0.79), 6MWD (AUC 0.66; 95% CI 0.58–0.74), PVR (AUC 0.58; 95% CI 0.49–0.67), and CI (cardiac index) (AUC 0.58; 95% CI 0.49–0.67, Figure 4A). The cut-off value of GGT levels was 53 U/L with a sensitivity of 66% and a specificity of 78%. As shown in Table 2, in GGT activity <53 U/L group, female patients had less 6MWD than male patients. However, in GGT activity ≥ 53 U/L group, male patients had higher PVR and *BMPR2* mutation proportion. In 192 patients with a GGT activity <53 U/L, 48 (25%) patients died, whereas 81 (65%) of the 124 patients died in GGT activity ≥ 53 U/L. Compared with patients in GGT activity <53 U/L group, the survival rate was significantly lower in patients with GGT activity ≥ 53 U/L group (Figure 4B). The 1- and 3-years survival estimates were 81 and 56%, respectively, in patients with GGT activity ≥ 53 U/L, and 93 and 85%, respectively, in patients with GGT activity <53 U/L (log-rank test, *p* < 0.001).

**Table 2:** Characteristics of study population in relation to GGT.

**Variables**	**GGT** **<53 U/L**		**GGT** **≥53 U/L**	
	**Male (*n* = 42)**	**Female (*n* = 150)**	**Male (*n* = 58)**	**Female (*n* = 66)**
Age, years	44 ± 20	38 ± 13	45 ± 18	41 ± 15
BMI, kg/m^2^	23 (20–25)	22 (20–25)	22 (20–24)	22 (21–25)
6MWD, meters^†^	430 (356–485)	385 (325–445)^*^	360 (299–468)	345 (284–418)
WHO FC, *n* (%)
Class I/II	17 (40)	55 (37)	12 (21)	13 (20)
Class III	25 (60)	87 (58)	38 (65)	49 (74)
Class IV	0 (0)	8 (5)	8 (14)	4 (6)
*BMPR2* mutation, n (%)^‡^	9 (35)	21 (22)	12 (40)	4 (11)^*^
NT–proBNP, ng/L	654 ± 851	886 ± 914	1,933 ± 1,725	1,614 ± 1,194
**Hemodynamics**				
mRAP, mmHg	5.4 ± 4.0	5.5 ± 4.1	8.8 ± 6.3	9.5 ± 5.8
mPAP, mmHg	58.1 ± 17.8	55.8 ± 14.1	62.0 ± 16.4	59 ± 12.7
PAWP, mmHg	8.0 ± 3.1	6.9 ± 3.3	8.7 ± 3.2	7.8 ± 3.1
CI, L/min/m^2^	2.7 ± 0.8	2.7 ± 0.7	2.3 ± 0.7	2.3 ± 0.7
PVR, Wood units	11.7 ± 5.4	12.9 ± 6.1	16.1 ± 7.0	14.5 ± 7.0^*^
S_V_O_2_, %	65 (58–70)	66 (59–71)	59 (53–67)	59 (52–65)
**Echocardiography**				
RA area (cm^2^)^§^	23 ± 11	20 ± 9	34 ± 16	28 ± 12

*Data were presented as numbers of patients (n, %), mean ± standard deviations or median (interquartile range)*.

* *Comparison between male and female, p < 0.05*.

† *Data on 6MWD was available from 275 individuals*.

‡ *Data on BMPR2 mutation measurement was available from 200 individuals*.

§ *RA area was measured in 222 individuals*.

*BMI, body mass index; BMPR2, bone morphogenetic protein receptor type 2; CI, cardiac index; GGT, gamma-glutamyltranspeptidase; mPAP, mean pulmonary arterial pressure; PAWP, mean pulmonary artery wedge pressure; mRAP, mean right atrial pressure; 6MWD, 6-minute walking distance; NT-proBNP, N-terminal pro B-type natriuretic peptide; PVR, pulmonary vascular resistance; S_v_O_2_, mixed venous oxygen saturation; WHO FC, World Health Organization functional class*.

Particularly, if GGT activity ≥ 53 U/L, male patients had significantly worse survival than female patients (Figure 4C). In GGT activity ≥ 53 U/L group, 1- and 3-years survival estimates was 74 and 48%, respectively, in male patients, and 88 and 64%, respectively, in female patients (log-rank test, *p* = 0.008). However, we did not find a different survival between females and males in GGT activity <53 U/L group (Figure 4D). In GGT activity <53 U/L group, the 1- and 3 years survival estimates were 93 and 83%, respectively, in male patients, and 93 and 86%, respectively, in female patients (log-rank test, *p* = 0.87).

### GGT in the Context of Other Markers of Adverse Prognosis

Univariate Cox proportional hazard analysis showed the risk of increased GGT activity to predict the mortality in all patients [hazard radio (HR) (95% CI), 5.37 (2.51–11.49), *p* < 0.001 in male; HR (95%CI), 2.44 (1.55–3.85), *p* < 0.001 in female, Table 3]. According to forward stepwise multivariate Cox proportional hazard analyses, the correlation between higher GGT activity and increased all-cause mortality adjusted by age [HR (95%CI), 5.26 (2.45–11.25), *p* < 0.001 in male; HR (95%CI), 2.42 (1.53–3.81), *p* < 0.001 in female] and then 6MWD, WHO functional classes, NT-proBNP [HR (95%CI), 4.08 (1.36–12.18), *p* = 0.012 in male; *p* = 0.127 in female]. Further adjustment for CI, S_V_O_2_, and PVR, patients in higher GGT activity had increased risk of mortality [HR (95%CI), 3.97 (1.33–11.84), *p* = 0.013 in male; *p* = 0.136 in female].

**Table 3:** Cox proportional hazard analysis for GGT levels on mortality in males and females.

	**Male (*****n*** **=** **105)**		**Female (*****n*** **=** **233)**	
	**HR (95% CI)**	***p*-value**	**HR (95% CI)**	***p*-value**
Model 0	5.37 (2.51–11.49)	<0.001	2.44 (1.55–3.85)	<0.001
Model 1	5.26 (2.45–11.25)	<0.001	2.42 (1.53–3.81)	<0.001
Model 2	4.08 (1.36–12.18)	0.012	1.87 (0.84–4.20)	0.127
Model 3	3.97 (1.33–11.84)	0.013	1.88 (0.82–4.31)	0.136

*Model 0, GGT levels were dichotomised at 53 U/L; Model 1, adjusted for age; Model 2, Model 1 plus 6-minute walking distance and World Health Organization functional class; Model 3, Model 2 plus pulmonary vascular resistance, mixed venous oxygen saturation and cardiac index*.

*CI, confidence interval; GGT, gamma-glutamyltranspeptidase; HR, Hazard Ratio*.

### Changes of GGT in Rehospitalization in Male

**Figure 5:**
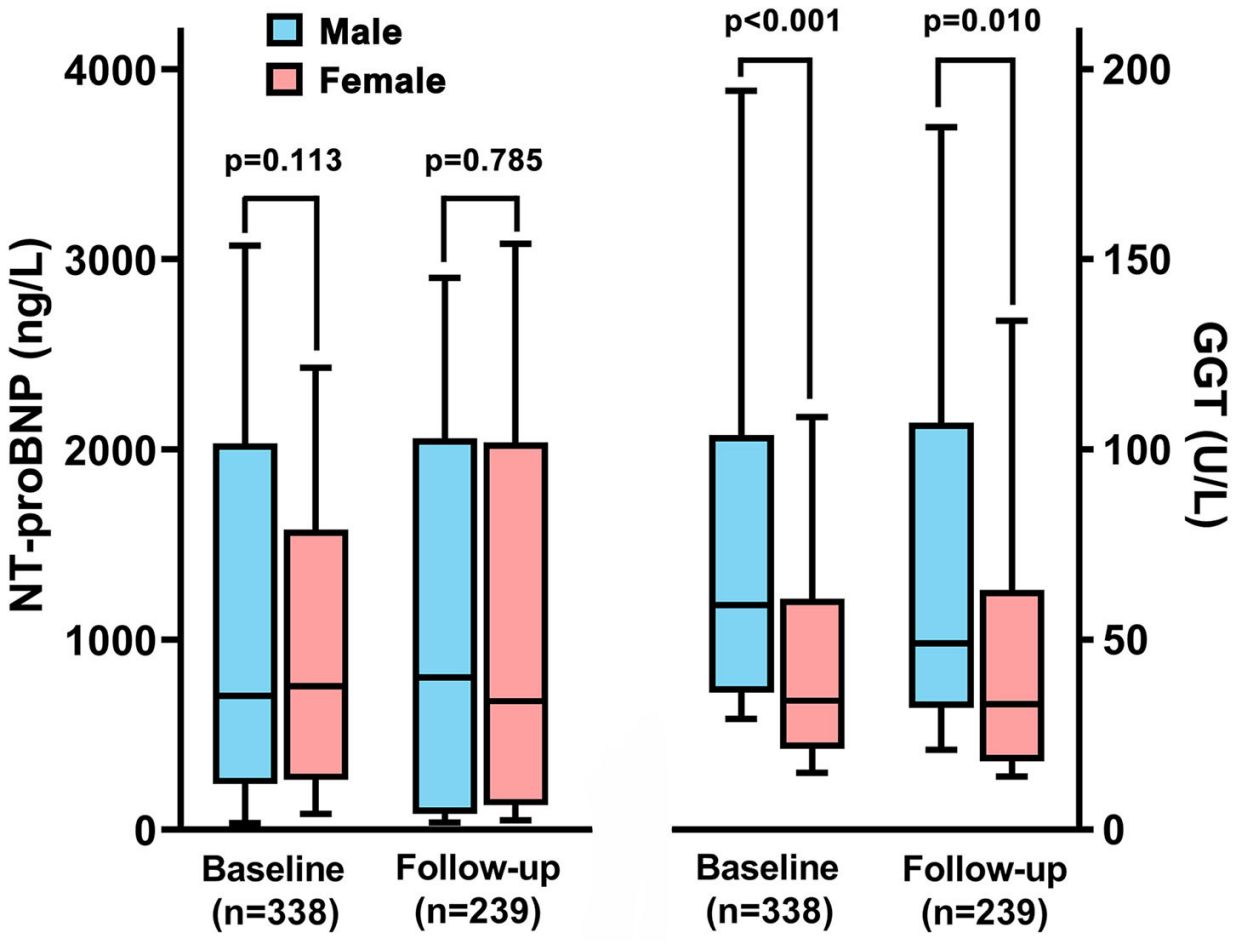
The changes of N-terminal pro B-type natriuretic peptide (NT-proBNP) and gamma-glutamyltranspeptidase (GGT) between male and female patients in baseline and rehospitalization.

During the follow-up interval, 239 patients (30% male patients) occurred rehospitalization, and 60% of them were due to clinical deterioration to change or add another targeted drug therapy Although there was no difference of GGT activity in total patients between baseline and rehospitalization, male patients still had higher value of GGT activity than females (86.9 ± 121.9 vs. 54.2 ± 61.8 U/L, *p* = 0.01, Figure 5). However, we did not find the significant difference of NT-proBNP in regard to sex different at follow-up, which was similar to the results of baseline.

## Discussion

Most CVD such as atherosclerosis, dilated cardiomyopathy, myocardial infarction and myocarditis occur predominantly in males, but in PAH the majority of patients are females (5, 24). However, not all of these diseases show that increased GGT activity is associated with poor outcomes in male patients (7, 9, 16). So far, it is unclear the prognostic value of GGT in different sex in patients with IPAH. In present study, we demonstrated that (a) serum GGT levels were higher in male patients compared with those in female patients; (b) male patients with *BMPR2* mutation had further elevated GGT levels than those of female patients; (c) GGT activity was significantly correlated with hemodynamic dysfunction and NT-proBNP in all patients; (d) male patients had worse survival compared with female patients in higher GGT activity group (e) serum GGT could be an independent predictor for all-cause mortality in male patients.

Some previous studies have reported the association between serum GGT and CVD mortality with sex differences. Haring et al. recruited 4160 subjects to confirm the prognostic value of GGT for cardiovascular events (16). They found GGT was only associated with the increased cardiovascular and all-cause mortality in males, but not in females. Accordingly, Strasak's team followed-up a healthy population of 76,113 Austrian males and females over 10 years (25). This study indicated that the longitudinal change of GGT activity > 9.2 U/L over 7 years was significantly correlated with increased cardiovascular-related mortality in males with less evident effects in females compared with stable GGT activity (−0.7 to 1.3 U/L). Interestingly, the sex difference in the association between GGT and cardiovascular events in Japanese patients is controversial (17). Japanese females had a stronger association of GGT with cardiovascular mortality and stroke than males. However, another Japanese study with 15 years follow-up demonstrated the CVD was significantly associate with females, but non-significant in males (26). The reason for this sex difference was unclear, and it might be partly accounted for by the difficulties in controlling the impacts of alcohol consumption in males (27). But in our cohort, we have excluded patients with alcohol consumption and hypercholesterolaemia and non-alcoholic fatty liver disease, et al. Therefore, further studies are needed to confirm whether serum GGT levels are correlated with mortality in IPAH patients with sex differences in a larger population.

Although our study did not directly determine the pathological mechanisms explaining the effect of GGT to sex differences for IPAH outcomes, some previous studies might have partially accounted for this phenomenon (27, 28). Elevated GGT activity might be a marker of antioxidant deficiency (27) and of improved oxidative stress in CVD (28) because of the high association with the traditional cardiovascular risk factors and possibly involved directly in the pathophysiology, and promotion of atherosclerosis by the generation of reactive oxygen species (27). Taguchi et al. found that serum GGT could reflect the antioxidant properties in healthy Japanese men (29). Interestingly, a research from Siqueira et al. showed that in female rats with PAH by ovariectomy, GGT could affect the oxidative stress and antioxidant system via estrogen (30). So, one of possible reasons was “estrogen withdrawal,” alleviating protective role of estrogen (E2) against oxidative damage (31). Another main mechanism involved that estrogen recused severe PH in rats by restoring lung and RV structure and function (32). Estrogen-induced recue of PH was associated with neoangiogenesis, suppression of inflammation and fibrosis (32). Therefore, we speculated that the lack of estrogen in male patients would increase the expression of superoxide radical and decrease lung and RV angiogenesis, which associated with the alteration of GGT level.

Moreover, our results showed that GGT activity had better correlation with NT-proBNP, RA area and hemodynamics in males, indicating abnormal GGT levels were consistent with the severity of PAH and RV dysfunction (1, 20). These results were similar with Zorlu' work, increased GGT activity was correlated with impaired hemodynamics and predicted early mortality in the patients with acute pulmonary embolism (33). A population-based research revealed that males had worse adaptive remodeling of RV against the increased afterload due to sex hormone-driven effects on RV dysfunction (3, 34). Therefore, we suggested that the values of GGT might response worse RV function in male patients compared with female patients.

We also found patients with *BMPR2* mutation had significantly elevated GGT activity in males compared with females, while those with non-mutation had no obvious sex difference. It might indicate that *BMPR2* signal was an important factor in GGT activity change in males. In our previous study, the overall survival difference of *BMPR2* mutation carriers compared with non-carriers was more obvious in males than females, due to the more complicated pathogenesis of PAH in female patients, where the impact of *BMPR2* mutation was greatly modified by other unknown factors (35). For instance, Fessel et al. found that oxidative stress might have a direct and crucial role in the pathway of *BMPR2*-mediated PAH in animal models (36). More severe oxidative stress in male patients with *BMPR2* mutation might be the reason for increased GGT activity (36). Another explanation was that *BMPR2* mutation contributed to poor RV function more directly in males, whereas it weakened in females (2). However, little is known about sex-specific differences in *BMPR2* expression and its potential effects in RV function in PAH (2). We supposed GGT activity reflected that male patients with *BMPR2* mutation were under “double strike” by vascular damage and hemodynamic abnormality.

## Study Limitations

There are several limitations in this study. First, this is a retrospectively study in a single center and the sample size is not large enough. Second, the follow-up assessments are not standardized and only part of patients have GGT value. It is different to further analyze the relationship between the change of GGT level and progression of disease. Then, although we used the age to adjust the survival analysis, age still influenced the activity of GGT and prognosis in all patients over time. Many patients did not perform a rehospitalization evaluation because of the long journey or expensive costs in therapy. Therefore, there were few patients having hemodynamics in follow-up interval. Finally, we do not consider the state of menstrual cyclus of women when blood was drawn. So, it is different to illustrate the influence of estrogen on GGT in sex difference.

## Conclusions

In summary, our study has demonstrated that serum GGT activity was significantly increased in male patients with IPAH compared with females. And in patients with *BMPR2* mutation, men also had higher serum GGT activity than women. Baseline GGT activity could reflect disease severity and predict a poor prognosis in male patients. In future, we need to further clarify the importance of GGT in the pathophysiology of PAH and its influence of sex differences, especially in male patients.

## Data Availability Statement

The data analyzed in this study is subject to the following licenses/restrictions: we will upload our dataset if reviewed. Requests to access these datasets should be directed to Gang-Hua Lu, 1653057@tongji.edu.cn.

## Ethics Statement

The studies involving human participants were reviewed and approved by Shanghai Pulmonary Hospital Ethics Committee (Number: K18-025). The patients/participants provided their written informed consent to participate in this study.

## Author's Note

The part of this study (<300 words) was accepted by the American Thoracic Society (ATS) as a poster presentation at their 2020 International Conference.

## Author Contributions

RZ and LW contributed to the study design, study conduct, supervision, scientific overview, data analysis, editing of the manuscript, and were also directly involved in the patients' recruitment and care. G-HL and S-GG contributed to patient enrolment, data analysis, scientific interpretation, drafting, and editing the original manuscript. CL, Q-HZ, RJ, and C-JL contributed to recruitment of participants, data collection, curation, and formal analysis. All authors have reviewed the manuscript, approved the final version for submission, participated in the design of the study, patient enrolment, and meet criteria for authorship.

## Conflict of Interest

The authors declare that the research was conducted in the absence of any commercial or financial relationships that could be construed as a potential conflict of interest.

## Abbreviations

BMI: body mass index
*BMPR2*: bone morphogenetic protein receptor type 2
BUN: blood urea nitrogen
CI: cardiac index
95% CI: 95% confidence interval
CVD: cardiovascular disease
GGT: gamma-glutamyltransferase
IPAH: idiopathic pulmonary arterial hypertension
mPAP: mean pulmonary arterial pressure
mRAP: mean right atrial pressure
6MWD: 6-minute walk distance
NT-proBNP: N-terminal pro B-type natriuretic peptide
PAH: pulmonary arterial hypertension
PAWP: pulmonary artery wedge pressure
PVR: pulmonary vascular resistance
RA: right atrial
RHC: right heart catheterization
RV: right ventricular
S_V_O_2_: mixed venous oxygen saturation
UA: uric acid
WHO FC: World Health Organization functional class.

## References

[1] Simonneau G Montani D Celermajer DS Denton CP Gatzoulis MA Krowka M. Haemodynamic definitions and updated clinical classification of pulmonary hypertension. Eur Respir J. (2019) 53:1801913. 10.1183/13993003.01913-2018 30545968 PMC6351336

[2] Docherty CK Harvey KY Mair KM Griffin S Denver N MacLean MR. The role of sex in the pathophysiology of pulmonary hypertension. Adv Exp Med Biol. (2018) 1065:511–8. 10.1007/978-3-319-77932-4_31 30051404

[3] Foderaro A Ventetuolo CE. Pulmonary arterial hypertension and the sex hormone paradox. Curr Hypertens Rep. (2016) 18:84. 10.1007/s11906-016-0689-7 27832457

[4] Austin ED Hamid R Hemnes AR Loyd JE Blackwell T Yu C BMPR2 expression is suppressed by signaling through the estrogen receptor. Biol Sex Differ. (2012) 3:6 10.1186/2042-6410-3-6 22348410 PMC3310853

[5] Batton KA Austin CO Bruno KA Burger CD Shapiro BP Fairweather D. Sex differences in pulmonary arterial hypertension: role of infection and autoimmunity in the pathogenesis of disease. Biol Sex Differ. (2018) 9:15. 10.1186/s13293-018-0176-8 29669571 PMC5907450

[6] Tofovic SP Jackson EK. Estradiol metabolism: crossroads in pulmonary arterial hypertension. Int J Mol Sci. (2019) 21:116. 10.3390/ijms21010116 31877978 PMC6982327

[7] Whitfield JB Gamma glutamyl transferase. Crit Rev Clin Lab Sci. (2001) 38:263–355. 10.1080/20014091084227 11563810

[8] Kunutsor SK Gamma-glutamyltransferase-friend or foe within? Liver Int. (2016) 36:1723–34. 10.1111/liv.13221 27512925

[9] Schiele F Guilmin AM Detienne H Siest G. Gamma-glutamyltransferase activity in plasma: statistical distributions, individual variations, and reference intervals. Clin Chem. (1977) 23:1023–8. 10.1093/clinchem/23.6.1023 15742

[10] Lee DS Evans JC Robins SJ Wilson PW Albano I Fox CS. Gamma glutamyl transferase and metabolic syndrome, cardiovascular disease, and mortality risk: the Framingham Heart Study. Arterioscler Thromb Vasc Biol. (2007) 27:127–33. 10.1161/01.ATV.0000251993.20372.40 17095717

[11] Lee DH Silventoinen K Hu G Jacobs DR Jr Jousilahti P Sundvall J. Serum gamma-glutamyltransferase predicts non-fatal myocardial infarction and fatal coronary heart disease among 28,838 middle-aged men and women. Eur Heart J. (2006) 27:2170–6. 10.1093/eurheartj/ehl086 16772340

[12] Ruttmann E Brant LJ Concin H Diem G Rapp K Ulmer H Gammaglutamyltransferase as a risk factor for cardiovascular disease mortality: an epidemiological investigation in a cohort of 163,944 Austrian adults. Circulation. (2005) 112:2130–7. 10.1161/CIRCULATIONAHA.105.552547 16186419

[13] Lee DH Ha MH Kim JH Christiani DC Gross MD Steffes M. Gammaglutamyltransferase and diabetes-a 4 year follow-up study. Diabetologia. (2003) 46:359–64. 10.1007/s00125-003-1036-5 12687334

[14] Jimba S Nakagami T Oya J Wasada T Endo Y Iwamoto Y. Increase in gamma-glutamyltransferase level and development of established cardiovascular risk factors and diabetes in Japanese adults. Metab Syndr Relat Disord. (2009) 7:411–8. 10.1089/met.2008.0082 19419267

[15] Wannamethee SG Lwnnon L Shaper AG The value of gamma-glutamyltransferase in cardiovascular risk prediction in men without diagnosded cardiovascular disease or diabets. Atherosclerosis. (2008) 201:168–75. 10.1016/j.atherosclerosis.2008.01.019 18378241

[16] Haring R Wallaschofski H Nauck M Dorr M Baumeister SE Volzke H Ultrasonographic hepatic steatosis incrases prediction of mortality risk from elevated serum gamma-glutamyl transpeptidase levels. Hepatology. (2009) 50:1403–11. 10.1002/hep.23135 19670414

[17] Hozawa A Okamura T Kadowaki T Murakami Y Nakamura K Hayakawa T. gamma-Glutamyltransferase predicts cardiovascular death among Japanese women. Atherosclerosis. (2007) 194:498–504. 10.1016/j.atherosclerosis.2006.08.058 17034795

[18] Otto CM. Heartbeat: biomarkers and pulmonary artery hypertension. Heart. (2016) 102:333–4. 10.1136/heartjnl-2015-309220 26869632

[19] Galie N Hoeper MM Humber M Torbicki A Vachiery JL Barbera JA Guidelines for the diagnosis and treatment of pulmonary hypertension: the task force for the diagnosis and treatment of pulmonary hypertension of the European Society of Cardiology (ESC) and the European Respiratory Society (ERS), endorsed by the International Society of Heart and Lung Transplatation (ISHLT). Eur Heart J. (2009) 30:2493–537. 10.1093/eurheartj/ehp297 19713419

[20] Galie N Humbert M Vachiery JL Gibbs S Lang I Torbicki A. 2015 ESC/ERS Guidelines for the diagnosis and treatment of pulmonary hypertension: The Joint Task Force for the Diagnosis and Treatment of Pulmonary Hypertension of the European Society of Cardiology (ESC) and the European Respiratory Society (ERS): Endorsed by: Association for European Paediatric and Congenital Cardiology (AEPC), International Society for Heart and Lung Transplantation (ISHLT). Eur Respir J. (2015) 46:903–75. 10.1183/13993003.01032-2015 26318161

[21] American Thoracic Society; American College of Chest Physicians ATS/ACCP Statement on cardiopulmonary exercise testing. Am J Respir Crit Care Med. (2003) 167:211–77. 10.1164/rccm.167.2.211 12524257

[22] Rudski LG Lai WW Afilalo J Hua L Handschumacher MD Chandrasekaran K. Guidelines for the echocardiographic assessment of the right heart in adults: a report from the American Society of Echocardiography endorsed by the European Association of Echocardiography, a registered branch of the European Society of Cardiology, and the Canadian Society of Echocardiography. J Am Soc Echocardiogr. (2010) 23:685–713. 10.1016/j.echo.2010.05.010 20620859

[23] Steinmetz J Schiele F Gueguen R Ferard G Henny J Periodic Health Examination Centers Laboratory Working Group. Standardization of gamma-glutamyltransferase assays by intermethod calibration. Effect on determining common reference limits. Clin Chem Lab Med. (2007) 45:1373–80. 10.1515/CCLM.2007.290 17924850

[24] Arnold AP Cassis LA Eghbali M Reue K Sandberg K. Sex hormones and sex chromosomes cause sex differences in the development of cardiovascular diseases. Arterioscler Thromb Vasc Biol. (2017) 37:746–56. 10.1161/ATVBAHA.116.307301 28279969 PMC5437981

[25] Strasak AM Kelleher CC Klenk J Brant LJ Ruttmann E Rapp K. Longitudinal change in serum gamma-glutamyltransferase and cardiovascular disease mortality: a prospective population-based study in 76,113 Austrian adults. Arterioscler Thromb Vasc Biol. (2008) 28:1857–65. 10.1161/ATVBAHA.108.170597 18617645 PMC2643843

[26] Li Y Iso H Cui R Murakami Y Yatsuya H Miura K. Serum gammaglutamyltransferase and mortality due to cardiovascular disease in Japanese men and women. J Atheroscler Thromb. (2016) 23:792–9. 10.5551/jat.32698 26875518 PMC7399270

[27] Bulusu S Sharma M. What does serum γ-glutamyltransferase tell us as a cardiometabolic risk marker? Ann Clin Biochem. (2016) 53:312–32. 10.1177/0004563215597010 26139450

[28] Cho HS Lee SW Kim ES Mo EY Shin JY Moon SD. Clinical significance of serum bilirubin and gamma-alugamyltransferase levels on coronary atherosclerosis assessed by multidetector computed tomography. Nutr Metab Cardiobasc Dis. (2015) 25:677–85. 10.1016/j.numecd.2015.03.014 26026212

[29] Taguchi C Kishimoto Y Kondo K Tohyama K Goda T. Serum gammaglutamyltransferase is inversely associated with dietary total and coffeederived polyphenol intakes in apparently healthy Japanese men. Eur J Nutr. (2018) 57:2819–26. 10.1007/s00394-017-1549-1 28988315

[30] Siqueira R Colombo R Conzatti A de Castro AL Carraro CC Tavares AMV. Effects of ovariectomy on antioxidant defence systems in the right ventricle of female rats with pulmonary arterial hypertension induced by monocrotaline. Can J Physiol Pharmacol. (2018) 96:295–303. 10.1139/cjpp-2016-0445 28854338

[31] Bellanti F Matteo M Rollo T Rosaro FD Greco P Vendemiale G. Sex hormones modulate circulating antioxidant enzymes: impact of estrogen therapy. Redox Biol. (2013) 1:340–6. 10.1016/j.redox.2013.05.003 24024169 PMC3757703

[32] Umar S Lorga A Matori H Nadadur RD Li JY Maltese F. Estrogen rescues preexisting severe pulmonary hypertension in rats. Am J Respir Crit Care Med. (2011) 184:715–23. 10.1164/rccm.201101-0078OC 21700911 PMC3208600

[33] Zorlu A Yucel H Bektasoglu G Turkdogan KA Eryigit U Sarikaya S. Increased gamma-glutamyl transferase levels predict early mortality in patients with acute pulmonary embolism. Am J Emerg Med. (2012) 30:908–15. 10.1016/j.ajem.2011.12.040 22386346

[34] Jacobs W van de Veerdonk MC Trip P de Man F Heymans MW Marcus JT. The right ventricle explains sex differences in survival in idiopathic pulmonary arterial hypertension. Chest. (2014) 145:1230–6. 10.1378/chest.13-1291 24306900 PMC4042511

[35] Liu D Wu WH Mao YM Yuan P Zhang R Ju FL. BMPR2 mutations influence phenotype more obviously in male patients with pulmonary arterial hypertension. Circ Cardiovasc Genet. (2012) 5:511–8. 10.1161/CIRCGENETICS.111.962209 22923421

[36] Fessel JP Flynn CR Robinson LJ Penner NL Gladson S Kang CJ. Hyperoxia synergizes with mutant bone morphogenic protein receptor 2 to cause metabolic stress, oxidant injury, and pulmonary hypertension. Am J Respir Cell Mol Biol. (2013) 49:778–87. 10.1165/rcmb.2012-0463OC 23742019 PMC3931097

