# Supplementary material for: Prognostic Value of Gamma-Glutamyltransferase in Male Patients With Idiopathic Pulmonary Arterial Hypertension
Source: Front Cardiovasc Med. 2020 Oct 23;7:580908. doi: 10.3389/fcvm.2020.580908 (PMC7644547; doi:10.3389/fcvm.2020.580908)
Supplement: Supplementary file 1 [file Data_Sheet_1.pdf]

Supplementary Information

Influence of Gamma-glutamyltransferase on Idiopathic Pulmonary Arterial Hypertension in Male Patients

Gang-Hua Lu[1†], Su-Gang Gong[2†], Chao Li[1], Qin-Hua Zhao[2], Rong Jiang[2], Ci-Jun Luo[2], Lan Wang[1,2,*], Rui Zhang[1,2,*]

[1]Tongji University School of Medicine, Shanghai, 200092, China;

[2]Department of Cardio-Pulmonary Circulation, Shanghai Pulmonary Hospital, Tongji University School of Medicine, Shanghai, 200433, China;

[*]**Co-correspondence:**

Dr. Rui Zhang, MD, E-mail: zgr1219@163.com;

and Dr. Lan Wang, MD, E-mail: wanglan198212@163.com;

Department of Cardio-Pulmonary Circulation, Shanghai Pulmonary Hospital, Tongji University School of Medicine, No. 507 Zhengmin Road, Shanghai, 200433, China; Telephone: +86-21-65115006; Fax: +86-21-55662767;

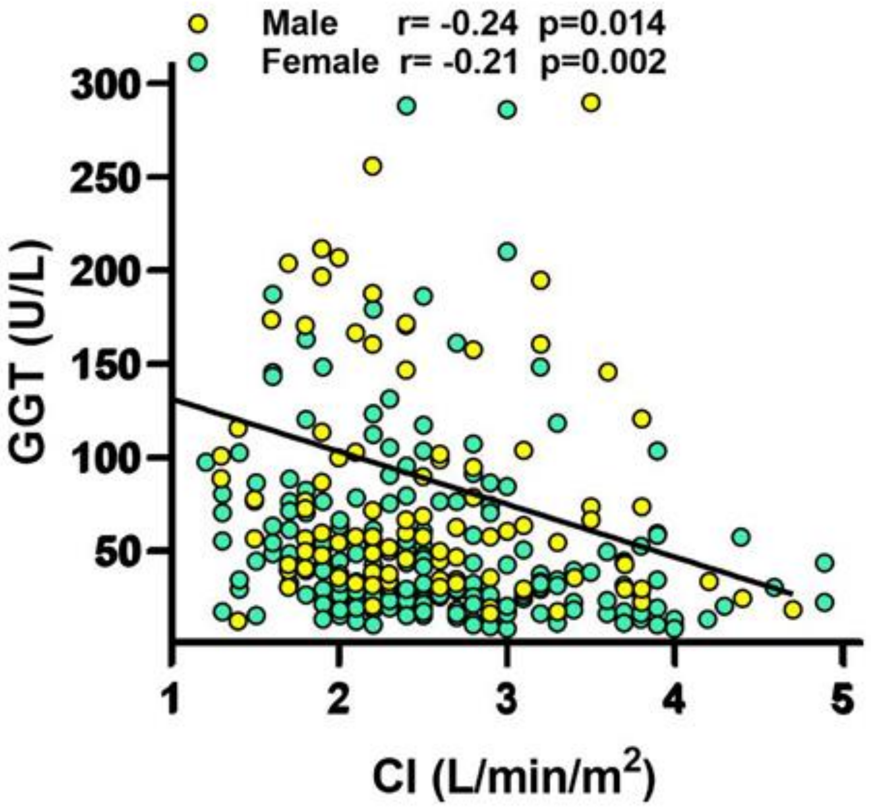

Supplemental Figure 1. Correlation between serum GGT activity and CI (r = -0.24, p = 0.014 in male; r = -0.21, p = 0.002 in female).

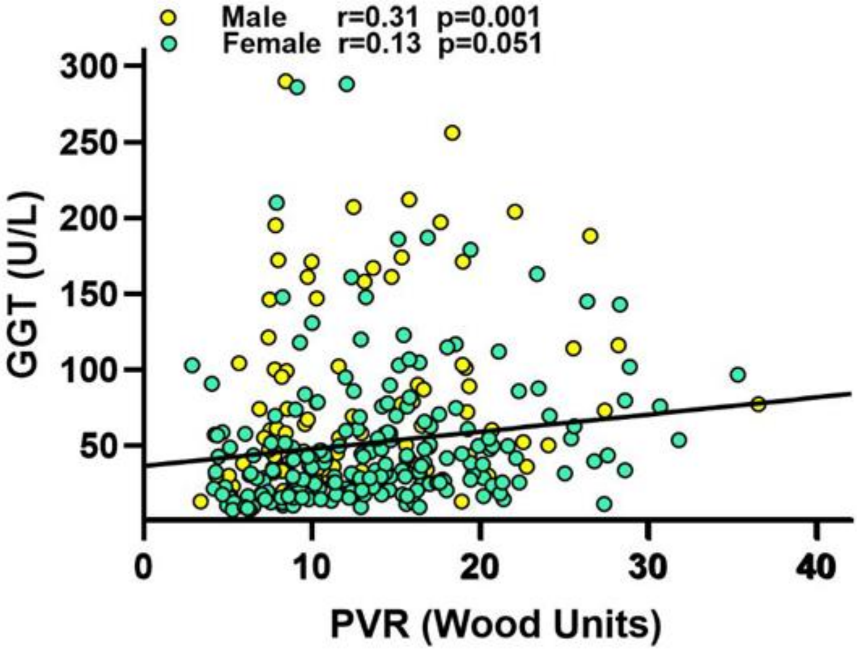

Supplemental Figure 2. Correlation between serum GGT activity and PVR (r = 0.31, p = 0.001 in male; r = 0.13, p = 0.051 in female).

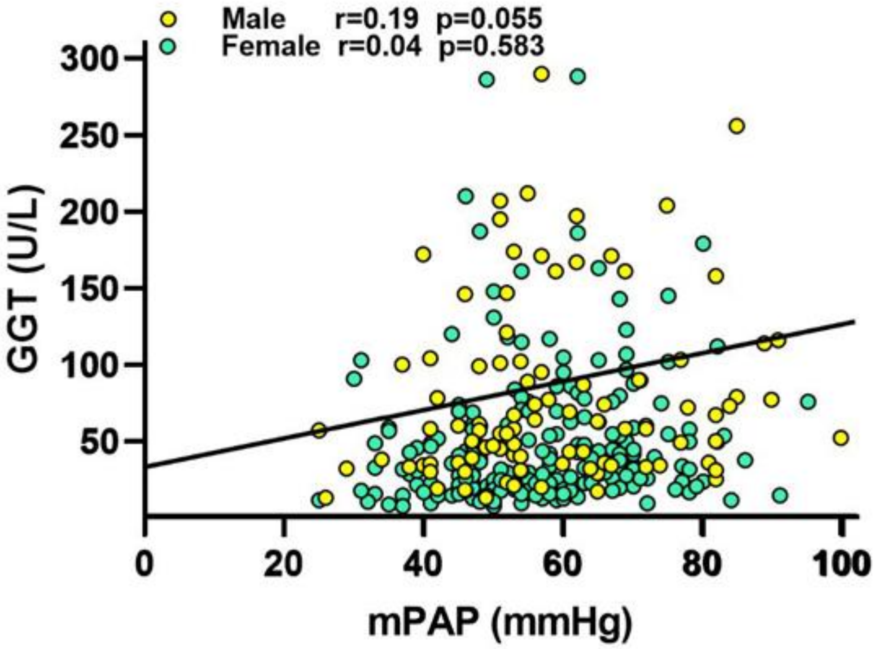

Supplemental Figure 3. Correlation between serum GGT activity and mPAP (r = 0.19, p = 0.055 in male; r = 0.04, p = 0.583 in female).

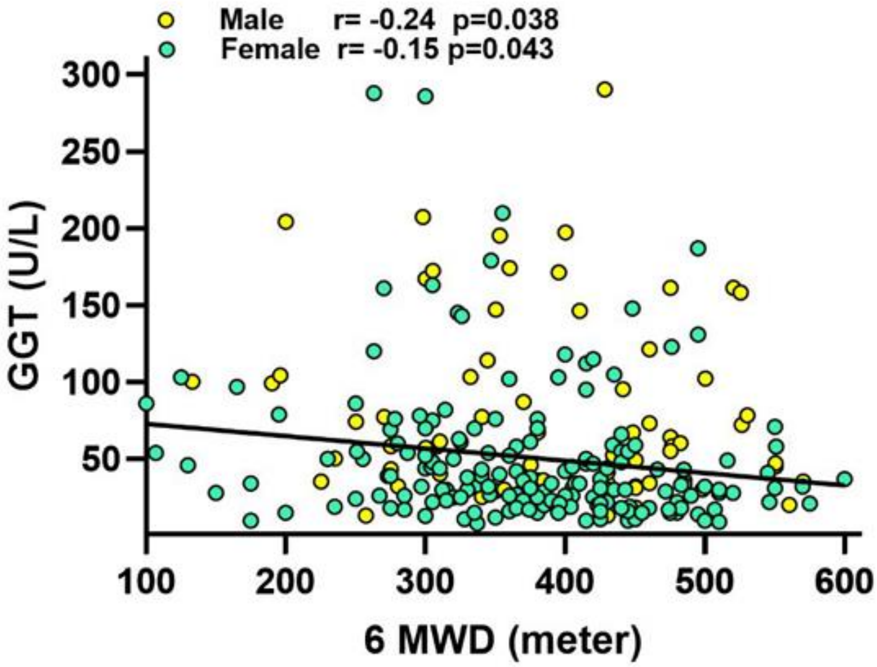

Supplemental Figure 4. Correlation between serum GGT activity and 6MWD (r = -0.24, p = 0.038 in male; r = -0.15, p = 0.043 in female).

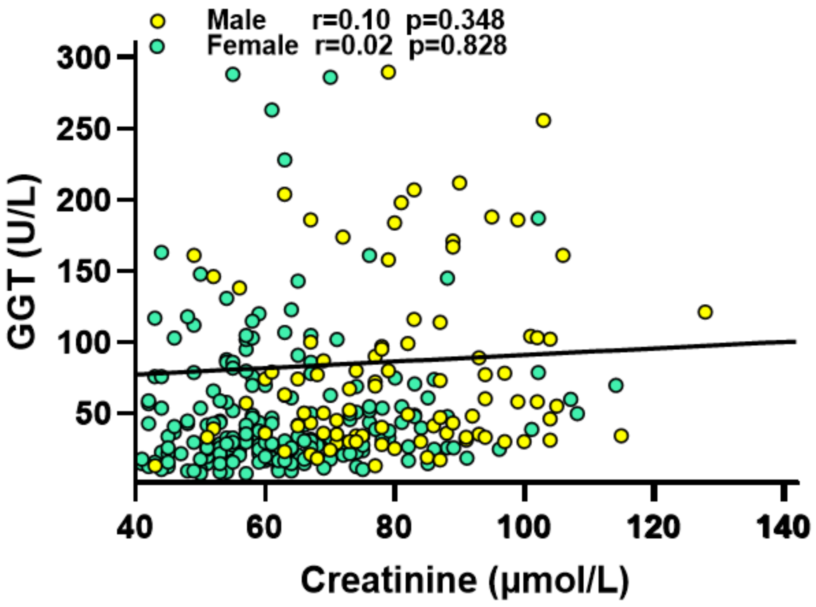

Supplemental Figure 5. Correlation between serum GGT activity and Creatinine (r = 0.10, p = 0.348 in male; r = 0.02, p = 0.828 in female).

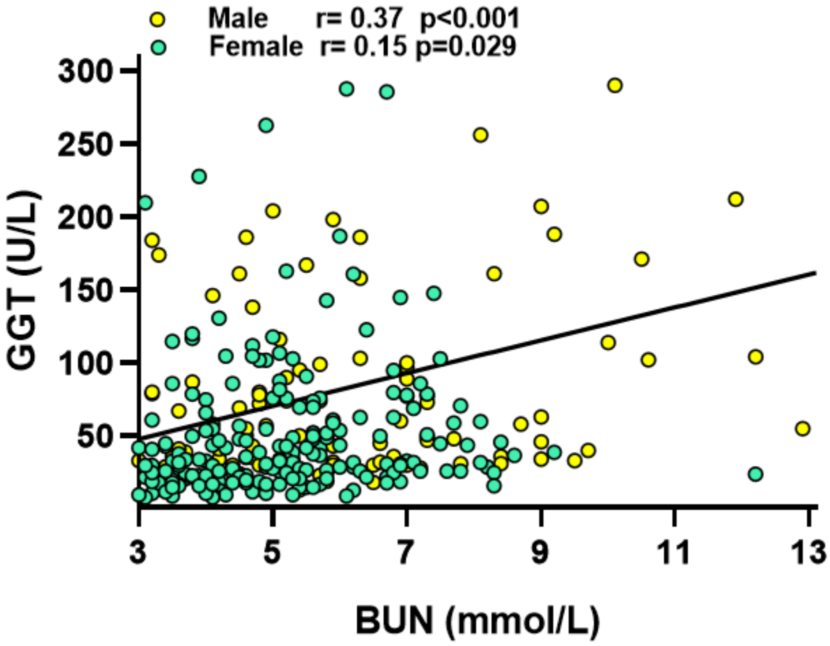

Supplemental Figure 6. Correlation between serum GGT activity and BUN (r = 0.37, p < 0.001 in male; r = 0.15, p = 0.029 in female).

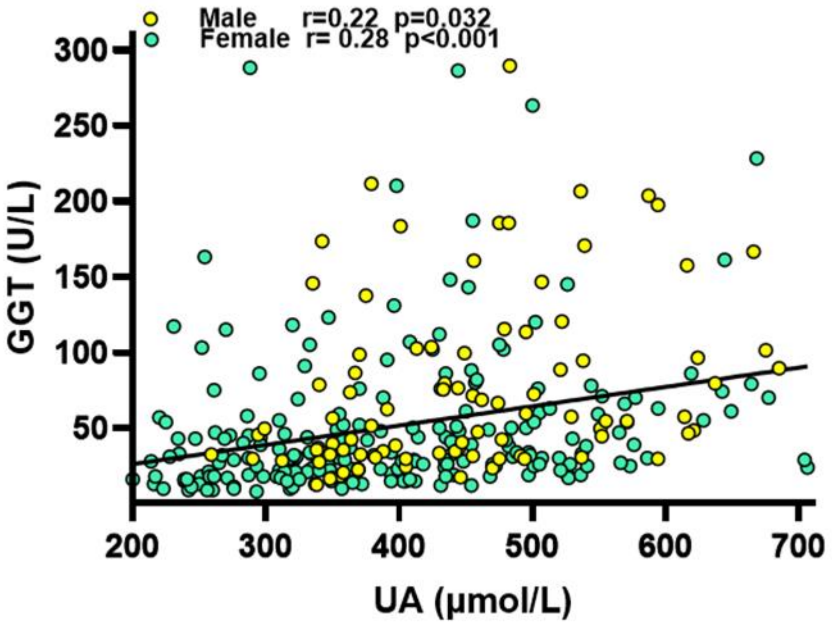

Supplemental Figure 7. Correlation between serum GGT activity and UA ($r = 0.22$, $p = 0.032$ in male; $r = 0.28$, $p < 0.001$ in female).

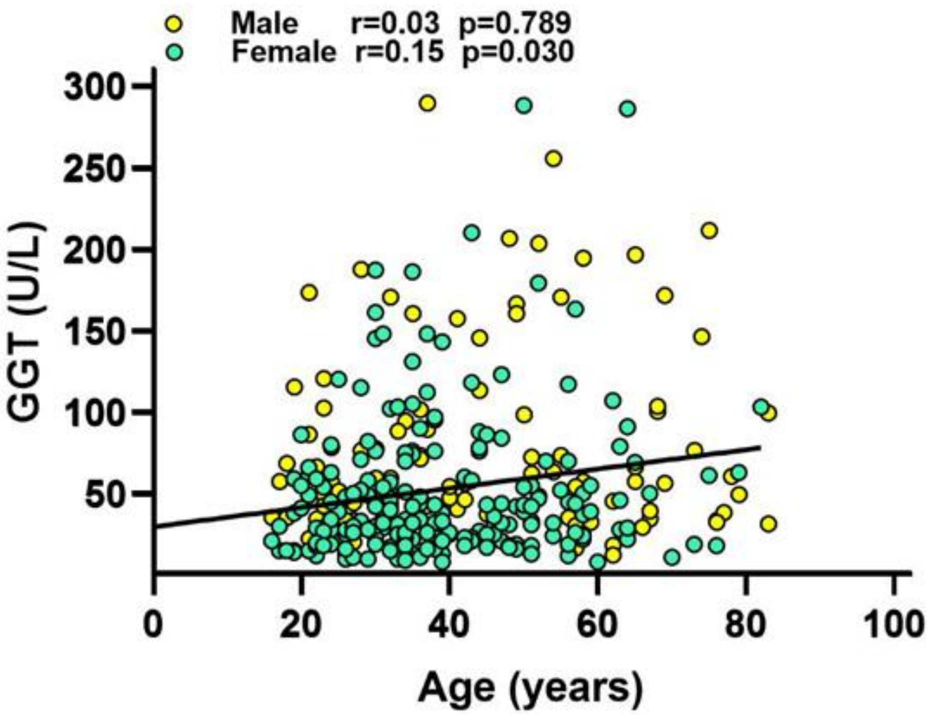

Supplemental Figure 8. Correlation between serum GGT activity and Age (r = 0.03, p = 0.789 in male; r = 0.15, p = 0.030 in female).

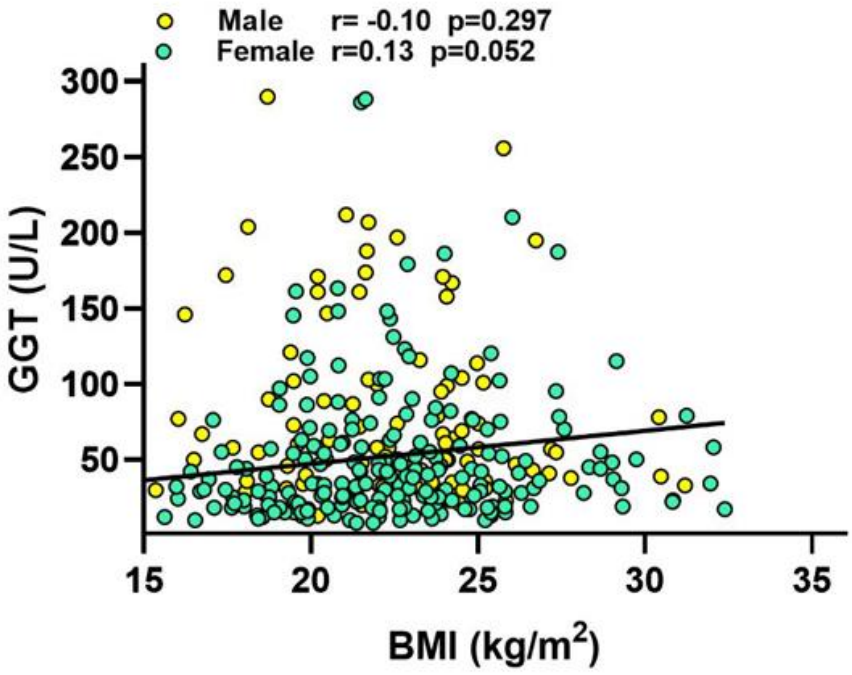

Supplemental Figure 9 Correlation between serum GGT activity and BMI (r = -0.10, p = 0.297 in male; r = 0.13, p = 0.052 in female).

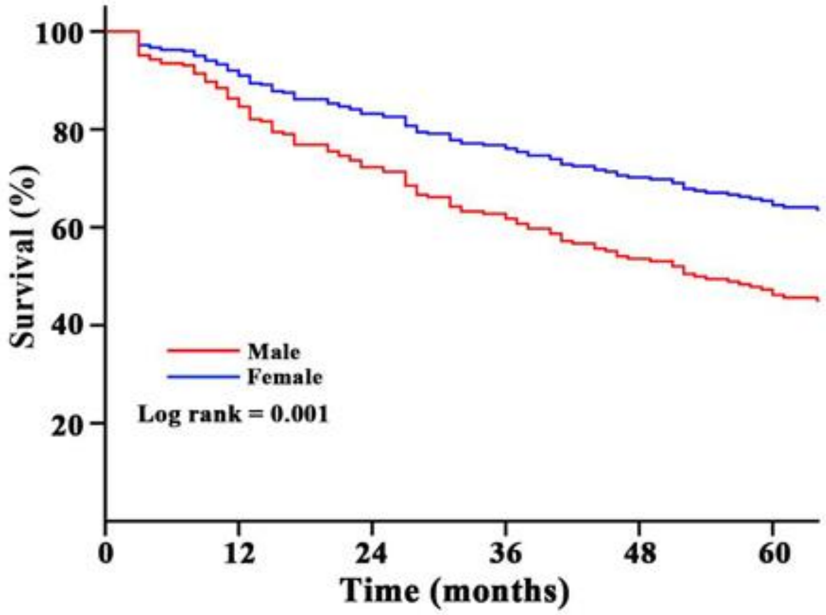

	Nunber of IPAH patients at risk (death or transplantation in %)					
Male	105 (0)	83 (17)	65 (27)	48 (36)	40 (43)	32 (48)
Female	233 (0)	190 (8)	159 (16)	131 (21)	117 (25)	95 (29)

Supplemental Figure 10. Kaplan-Meier survival curves according to the different gender.
